# Icaritin, an inhibitor of beta-site amyloid cleaving enzyme-1, inhibits secretion of amyloid precursor protein in APP-PS1-HEK293 cells by impeding the amyloidogenic pathway

**DOI:** 10.7717/peerj.8219

**Published:** 2019-12-10

**Authors:** Fei Feng, Yuanyuan Li, Nanqu Huang, Yong Luo

**Affiliations:** 1 Department of Neurology, The First People’s Hospital of Zunyi and Third Affiliated Hospital of Zunyi Medical University, Zunyi, Guizhou, China; 2 National Drug Clinical Trial Institution, The First People’s Hospital of Zunyi and Third Affiliated Hospital of Zunyi Medical University, Zunyi, Guizhou, China

**Keywords:** Alzheimer’s disease, Icaritin, Amyloid-β peptide, Beta-site amyloid cleaving enzyme 1, Presenilin-1, Amyloid precursor protein

## Abstract

**Background:**

Icaritin (ICT) is a prenylflavonoid derivative from *Epimedium brevicornum Maxim*. ICT has been shown to have neuroprotective effects. We investigate how ICT affects secretion of amyloid precursor protein (APP).

**Methods:**

We exposed APP-PS1-HEK293 cells to ICT to investigate its effect on beta-site amyloid cleaving enzyme (BACE)1. Cell viability was evaluated by MTT and lactate dehydrogenase (LDH) assays. The half-maximal inhibitory concentration (IC_50_) of ICT for BACE1 was measured using fluorescence resonance energy transfer. Effects of ICT on the mRNA expression of APP were assessed by quantitative polymerase chain reaction, and protein expression was measured by western blotting and immunofluorescence.

**Results:**

Icaritin inhibited BACE1 activity and IC_50_ was 5.70 ± 1.09 μM. Compared with the control group, at ICT concentrations of 5 μM and 10 μM, the viability increased and LDH leakage decreased in APP-PS1-293 cells. Also, mRNA expression of A disintegrin and metalloproteinase domain-containing protein 10 (ADAM10) increased, while that of BACE1 and presenilin-1 (PS1) decreased, upon ICT treatment. Western blotting and immunofluorescence confirmed that protein expression of ADAM10, BACE1 and PS1 showed the same trend. Expression of the APP fragments sAPPβ and C-terminal fragment β decreased, while that of sAPPα increased, upon ICT treatment. Expression of amyloid β peptides in APP-PS1-HEK293 cells was lower in ICT-treated groups compared with that in the control group.

**Conclusions:**

Icaritin, as a BACE1 inhibitor, inhibits APP secretion in APP-PS1-HEK293 cells by impeding the amyloidogenic pathway.

## Introduction

Alzheimer’s disease (AD) is a degenerative disease of the central nervous system characterized by progressive cognitive dysfunction and behavioral impairment. According to a report from Alzheimer’s Disease International, there are ~9.5 million dementia patients in China currently and this number may exceed 16 million by 2030 (Alzheimer’s Disease International, 2016). Due to a lack of efficacious therapeutic drugs, AD will be a major public health problem in China in the future. The exact etiology of AD is incompletely understood. Amyloid-β peptides (AβPs) are generated through sequential cleavage of amyloid precursor protein (APP) by beta-site amyloid cleaving enzyme (BACE)1 and γ-secretase (presenilin-1 (PS1)), which play pivotal roles in AD pathogenesis (Querfurth & LaFerla, 2010). However, a series of clinical trials on AβPs have failed, and research on AD has faltered (Voytyuk, De Strooper & Chávez-Gutiérrez, 2018).

The Chinese pharmaceutical chemist and 2015 Nobel Prize Laureate Youyou Tu and her research team studied artemisinin extracted from the stems and leaves of *Artemisia annua L*. They found that artemisinin can be used to treat malaria. Therefore, finding treatment for intractable diseases such as AD using phytomedicine could be a viable strategy. Icaritin (ICT) is a prenylflavonoid derivative from *Epimedium brevicornum Maxim*. The molecular weight of ICT is 386.4, it is soluble in fat, and can pass through the blood–brain barrier readily. ICT has been shown to enhance proliferation of mesenchymal stem cells (Lim et al., 2018), attenuate oxidative stress and the neuronal toxicity induced by AβPs (Wang et al., 2007; Wu et al., 2014). Furthermore, natural flavonoids can inhibit BACE-1 activity and reduce the level of secreted AβPs. Therefore, ICT could be an efficacious drug against AD.

**Figure 1:**
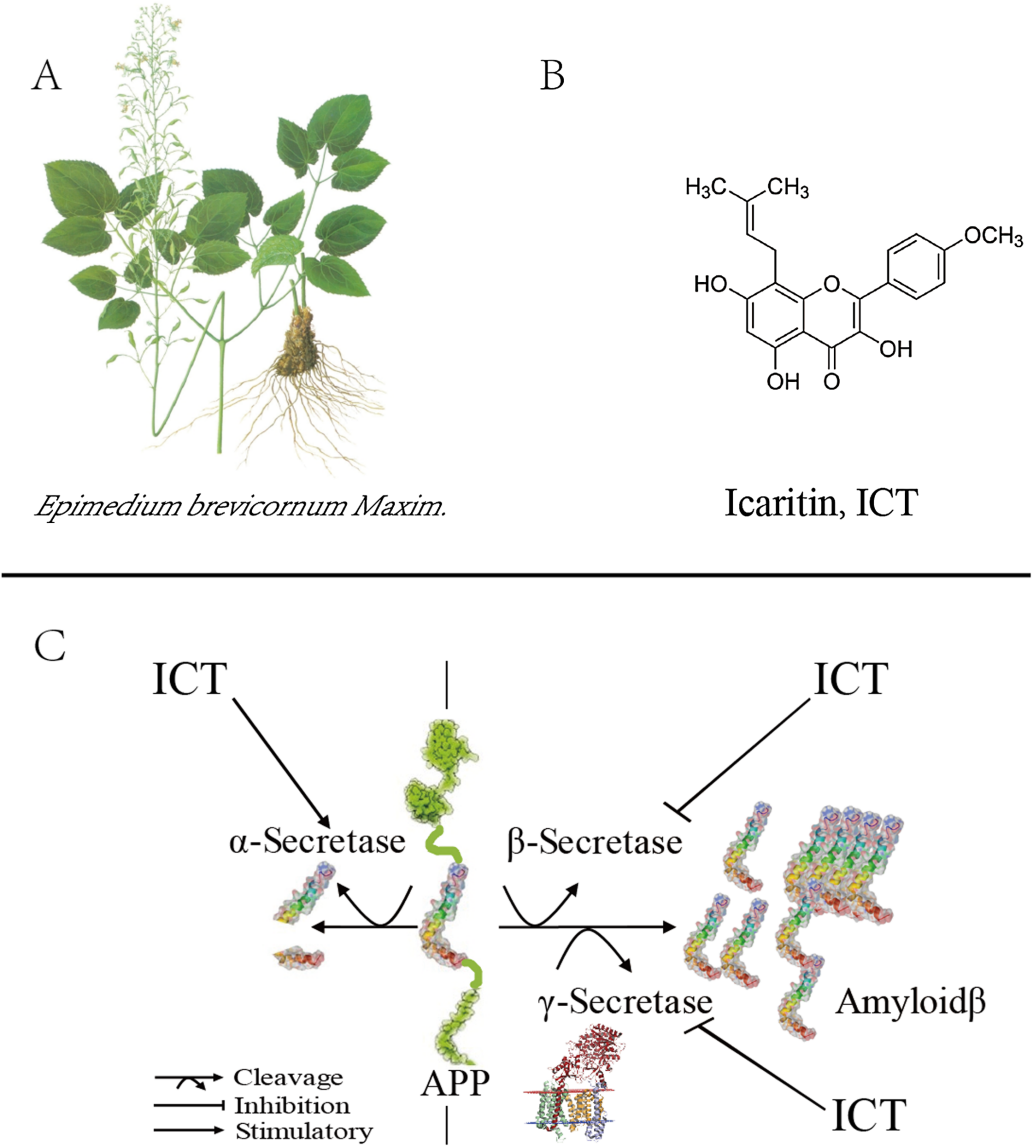
Role of ICT in the metabolic pathway of amyloid precursor proteins (schematic). (A) The plant is *Epimedium brevicornum Maxim*; (B) the chemical structure of ICT; (C) role of ICT in the metabolic pathway of amyloid precursor proteins (schematic).

Previously, we demonstrated that ICT can improve memory impairment in a rat model of AD by reducing AβP accumulation (Feng et al., 2017). To further explore the regulatory mechanism of ICT on AβP accumulation (Fig. 1), we designed a study to probe how ICT modulates expression of BACE1 and γ-secretase, and its potential neuroprotective effects, in vitro.

## Materials and Methods

### Materials

APP-PS1-HEK293 cells were purchased from Shanghai Enzyme-linked Biotechnology (Shanghai, China). ICT (purity >98.3%) was obtained from Nanjing Zelang Medical Technologies (Nanjing, China). 3-(4, 5-dimethylthiazol-2-yl)-2, 5-diphenyltetrazolium bromide (MTT) was purchased from Biosharp (Hefei, China). A lactate dehydrogenase (LDH) assay kit was obtained from Beyotime (Shanghai, China).

An electrochemiluminescence (ECL) kit was purchased from Thermo Fisher (Waltham, MA, USA). A BACE1 Fluorescence Resonance Energy Transfer (FRET) Assay kit was obtained from Panvera (Madison, WI, USA). A PrimeScript™ RT Reagent kit and SYBR™ Premix Ex Taq were purchased from TaKaRa Biotechnology (Shiga, Japan). Antibodies against anti-BACE1, anti-presenilin 1, anti-AβP_1–40_, anti-AβP_1–42_, anti-glyceraldehyde 3-phosphate dehydrogenase (GAPDH), and goat anti-rabbit immunoglobulin (Ig)G H&L (DyLight 488) were obtained from Abcam (Cambridge, UK). Anti-C-terminal fragment β (CTFβ) antibody was purchased from Biolegend (San Diego, CA, USA). Anti-sAPPβ (6A1) antibody and anti-sAPPa (2B3) antibody were obtained from Immuno-Biological Laboratories (Fujioka-Shi, Japan). Horseradish peroxidase-conjugated goat anti-mouse or anti-rabbit IgG antibody was purchased from Abmart (Shanghai, China).

### BACE1 inhibition assay

The BACE1 inhibition assay was executed using a BACE1 FRET kit according to manufacturer instructions. A stock solution of ICT in dimethyl sulfoxide was prepared (20 mmol/L). The sample was diluted further in assay buffer to prepare a 3× concentration of test compounds. A total of 10 μL of BACE1 substrate (a new “red” FRET peptide substrate based on the “Swedish” mutant, 3× concentration) was added to 10 μL of 3× concentration of each test compound in separate wells of a black 96 well microplate and mixed gently. Then, 10 μL of 3× BACE1 was added to each well to start the reaction. Plates were incubated for 90 min at 25 °C in the dark. Then, 10 μL of sodium acetate was added to each well to stop the reaction. Finally, fluorescence measurements were undertaken with a multimode microplate reader (Multiskan™ GO; Thermo Scientific, Waltham, MA, USA) at an excitation wavelength of 545 nm and emission wavelength of 585 nm. The half-maximal inhibitory concentration (IC_50_) was calculated by plotting the obtained relative fluorescence unit per hour (RFU/h) against the logarithmic of the inhibitor concentration. The measured inhibition data were analyzed in Prism 7 (GraphPad, La Jolla, CA, USA) by nonlinear regression (curve fitting).

### Cell culture

APP-PS1-HEK293 cells subcultured by 1:3, generations were maintained in Dulbecco’s modified Eagle’s medium (DMEM)/high glucose (SH30022.01B; Hyclone, Logan, MT, USA) supplemented with 10% fetal bovine serum (04-001-1A; Hyclone, Logan, MT, USA) and penicillin–streptomycin (SV30010; Hyclone, Logan, MT, USA) at 37 °C in a humidified atmosphere of 5% CO_2_.

### Cell viability by the MTT and LDH assays

Cell viability was determined by MTT and LDH assays. Briefly, APP-PS1-HEK293 cells cultured in 96 well plates (5,000/well) were treated with different concentrations of ICT for 16 h. The medium was removed and 50 μL of MTT reagent (0.5 mg/mL) in DMEM was added to each well. Then, cells were incubated for 3 h at 37 °C. After dissolving formazan crystals with 150 μL of dimethyl sulfoxide, absorbance at 570 nm was measured on a microplate reader (Multiskan™ GO; Thermo Scientific, Waltham, MA, USA). An LDH assay kit was used to evaluate injury/damage to cells according to manufacturer protocols, and absorbance at 490 nm was measured.

### Quantitative polymerase chain reaction

Total RNA from cultured cell samples were extracted using TRIzol™ Reagent (Sigma–Aldrich, St. Louis, MD, USA) through a series of rinse, elution, and centrifugation steps. RNA samples were reverse-transcribed using the PrimeScript RT Reagent kit (RR047A; TaKaRa Biotechnology, Shiga, Japan). SYBR Premix Ex Taq (RR820A; TaKaRa Biotechnology, Shiga, Japan) was used following manufacturer guidelines for measurement of mRNA expression of *BACE1*, *PS1*, and α-*Secretase*. All primer sequences are summarized in Table 1. Fluorescence signals were amplified and detected using a StepOnePlus™ sequence detector (Thermo Scientific, Waltham, MA, USA) and analyzed with Nanodrop 2000 (Thermo Scientific, Waltham, MA, USA). The cycle threshold (Ct) for each sample was averaged from triplicates. The 2^−ΔΔCt^ approach was used whereby fluorescence signals were normalized to the corresponding housekeeping gene *GAPDH*.

**Table 1:** The primer sequences used in the study.

Primer	Sequence (5′–3′)
BACE1 forward	GCGGGAGTGGTATTATGAAGTG
BACE1 reverse	CCACGATGCTCTTGTCATAGTT
PS1 forward	GAGCCCTGCACTCAATTCT
PS1 reverse	CCAGGCATGGATGACCTTATAG
α-Secretase forward	CAGGAAGCTCTGGAGGAATATG
α-Secretase reverse	GAGACTTTGGGAGGTACATGAG
GAPDH forward	GATGCTGGTGCTGAGTATGT
GAPDH reverse	GCGGAGATGATGACCCTTT

### Western blotting

APP-PS1-HEK293 cell lysates were prepared. Briefly, after 30 min of incubation with lysis buffer at 4 °C, insoluble material was removed by centrifugation at 12,000 rpm for 30 min at 4 °C. The protein concentration of the lysate was determined using a bicinchoninic acid protein assay kit (GK5012; Generay, Shanghai, China). Equal amounts (10 μg) of each sample were separated by sodium dodecyl sulfate-polyacrylamide gel electrophoresis (P0012AC; Beyotime, Shanghai, China) and transferred to polyvinylidene difluoride (PVDF) membranes (IPVH00010; Merck, Kenilworth, NJ, USA). PVDF membranes were blocked for 1 h in a 5% (w/v) fat-free milk solution in TBST (Tris buffer containing 0.5% (v/v) Tween-20). PVDF membranes were incubated overnight at 4 °C in 5% (w/v) bovine serum albumin solution in TBST with antibodies (all purchased from Abcam) against BACE1 antibody (1:1,000 dilution; ab2077), anti-PS1 (1:1,000; ab76083), anti-A disintegrin and metalloproteinase domain-containing protein 10 (ADAM10; 1:1,000; ab1997), anti-AβP_1–40_ (1:1,000; ab20068), and anti-GAPDH (1:1,000; ab8245). PVDF membranes were washed thrice (10 min each time) by TBST and incubated with horseradish peroxidase-conjugated goat anti-mouse or anti-rabbit IgG secondary antibodies (1:5,000; Abmart, Berkeley Heights, NJ, USA). Then, PVDF membranes were washed with TBST thrice and visualized using the ECL kit. Band intensity was quantified with Quantity One (Bio-Rad Laboratories, Hercules, CA, USA).

### Immunofluorescence

After treatment with different concentrations of ICT for 16 h, cells plated on coverslips were fixed with methanol for 20 min at 4 °C. They were permeabilized with 0.1% Triton X-100 in phosphate-buffered saline for 5 min at room temperature, and treated with blocking medium (5% bovine serum albumin in phosphate-buffered saline) for 30 min. Then, they were incubated with antibodies against anti-BACE1 (1:1,000 dilution), anti-PS1 (1:1,000), and anti-ADAM10 (1:1,000) at room temperature for 2 h. Immune-reacted primary antibody was detected following 1 h incubation in the dark at room temperature with a secondary antibody conjugated with Dylight 488 (1:1,000 dilution). Cells were further stained with 4′, 6-diamidino-2-phenylindole (C1002; Beyotime) for 2 min in the dark at room temperature and washed. Then, they were placed onto microscope slides in mounting medium. Observations were carried out using a fluorescence microscope (BX43F; Olympus, Tokyo, Japan).

### Statistical analyses

Data are the mean ± SEM from at least three independent experiments. Comparisons of more than two groups were made with one-way ANOVA, followed by Dunnett’s post hoc test. SPSS v22.0 (IBM, Armonk, NY, USA) was employed for statistical analyses *P* < 0.05 was considered significant.

## Results

### IC_50_ of ICT for BACE1

**Figure 2:**
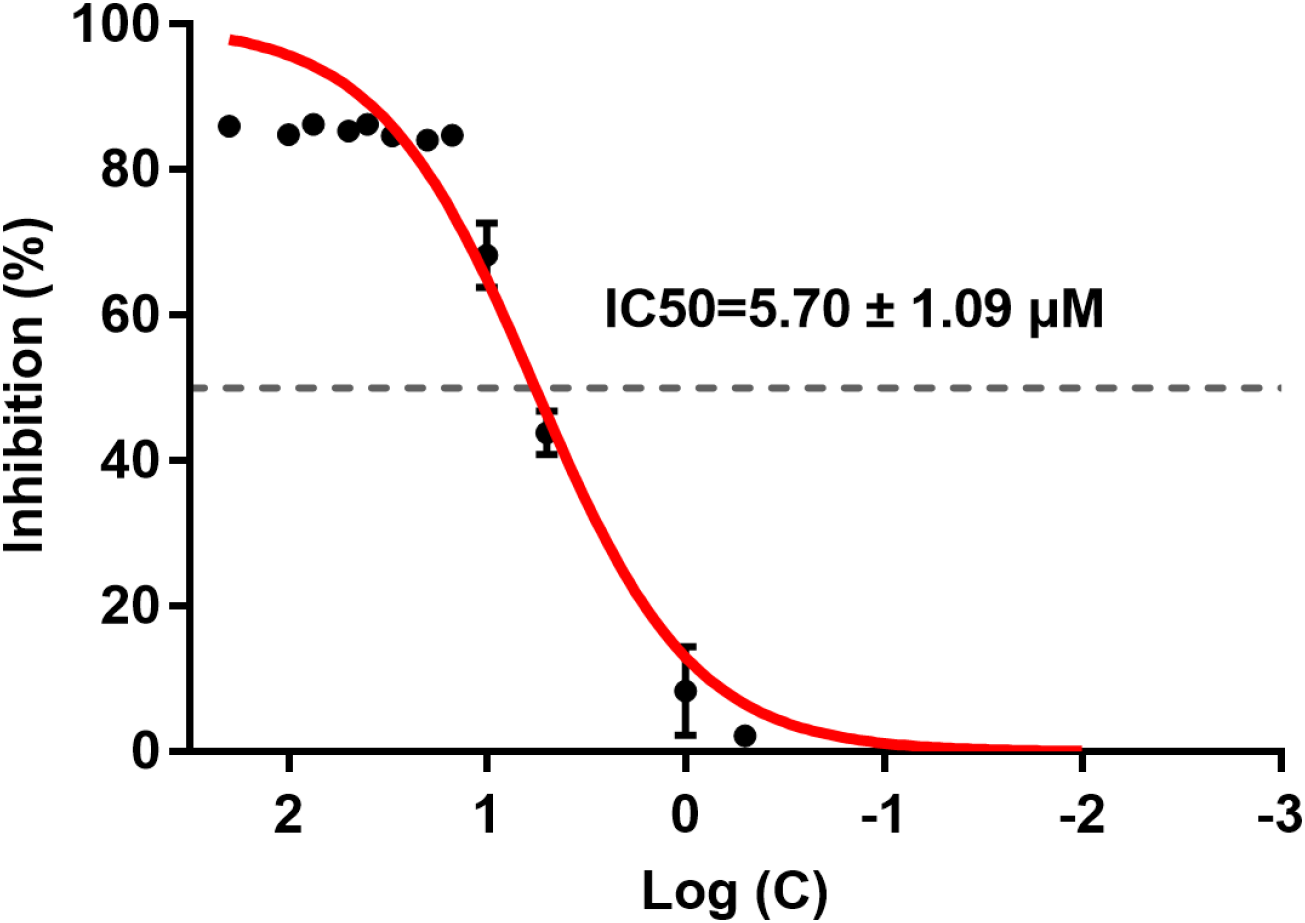
IC_50_ of ICT for BACE1. The BACE1 inhibition assay was executed using a BACE1 FRET kit. IC_50_ values were calculated by plotting the obtained relative fluorescence unit per hour (RFU/h) against the logarithmic of the inhibitor concentration. Mean ± SEM, *n* = 3.

ICT inhibited BACE1 activity and the IC_50_ was 5.70 ± 1.09 μM (Fig. 2). ICT inhibition was like that seen for other flavonoids, such as quercetin and apigenin (Sathya et al., 2012).

### Protective effect of ICT in APP-PS1-293 cells

**Figure 3:**
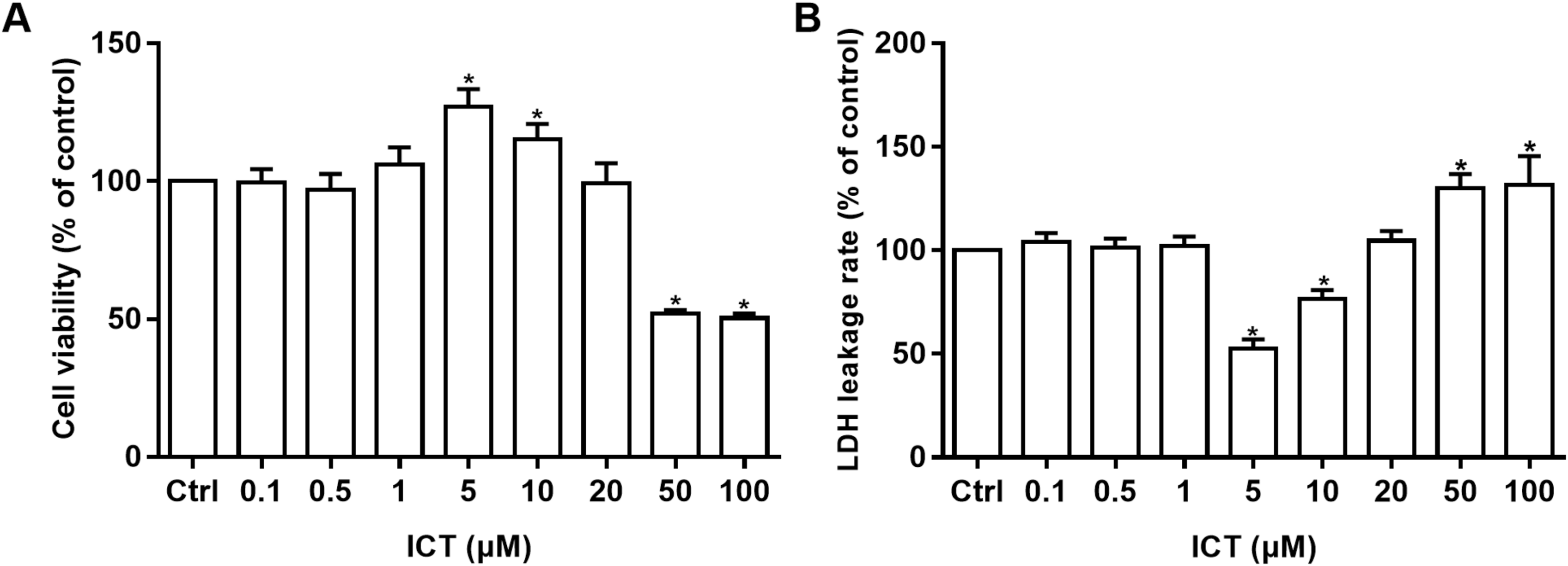
MTT and LDH assays in APP-PS1-293 cells. Effects of ICT (0.1, 0.5, 1, 5, 10, 20, 50, 100 μM) on (A) cell viability by the MTT assay and (B) LDH leakage (*P < 0.05 vs. control, mean ± SEM, *n* = 3).

Cell viability was measured by the MTT assay and cell-membrane damage was determined according to LDH leakage. These parameters were used to ascertain the neuroprotective effects of ICT in APP-PS1-293 cells. Compared with the control group, at ICT concentrations of 5 μM and 10 μM, the viability increased and LDH leakage decreased in APP-PS1-293 cells. These results suggested a neuroprotective effect of ICT in APP-PS1-293 cells at concentrations of 5 μM and 10 μM (Fig. 3).

### Effect of ICT on the amyloidogenic pathway of APP in APP-PS1-HEK293 cells

To investigate whether the amyloidogenic pathway is involved in the protective effects of ICT in APP-PS1-HEK293 cells, we detected some of the mRNA and proteins involved in this pathway.

**Figure 4:**
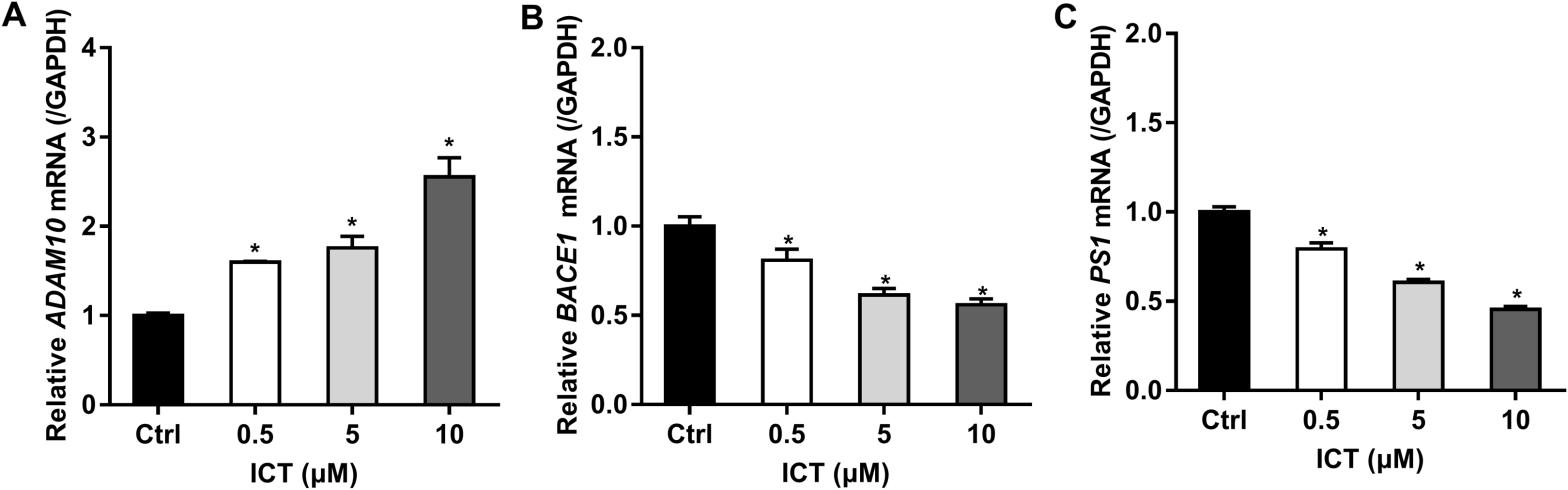
Effect of ICT (0.5, 5, 10 μM) on mRNA expression of *ADAM10*, *BACE1* and *PS1* in APP-PS1-HEK293 cells. (A) *ADAM10* mRNA; (B) *BACE1* mRNA; (C) *PS1* mRNA. mRNA expression of targets was normalized to that of GAPDH (*P < 0.05 vs. control, mean ± SEM, *n* = 3).

**Figure 5:**
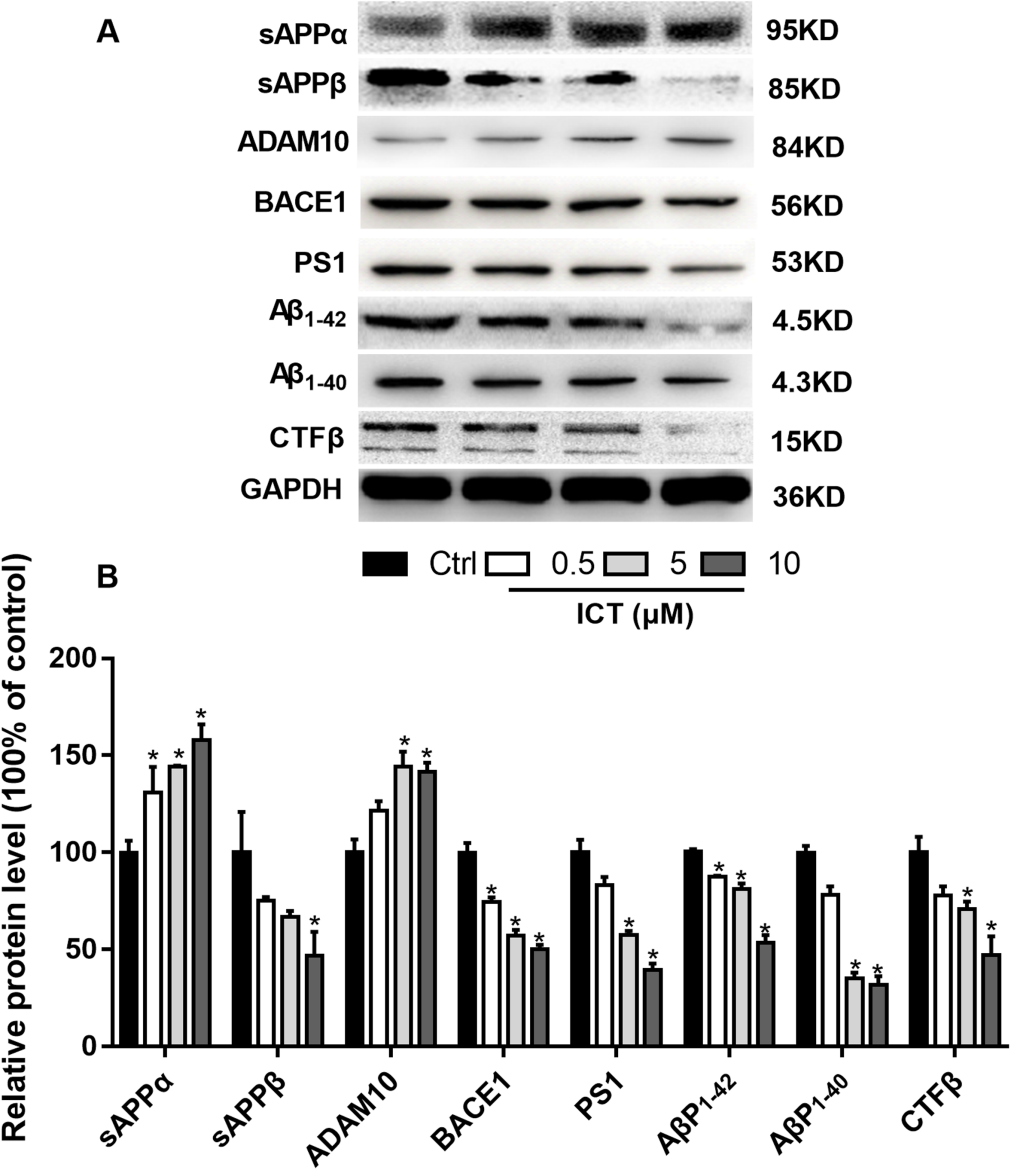
Effects of ICT on protein expression of the APP metabolic pathway in APP-PS1-HEK293 cells. (A) Representative bands of sAPPα, sAPPβ, ADAM10, BACE1, PS1, AβP_1–42_, AβP_1–40_, CTFβ and GAPDH; (B) relative protein expression of sAPPα, sAPPβ, ADAM10, BACE1, PS1, AβP_1–42_, AβP_1–40_, CTFβ and GAPDH. The relative absorbance was normalized to that of GAPDH. *P < 0.05 vs. control, mean ± SEM, *n* = 3.

**Figure 6:**
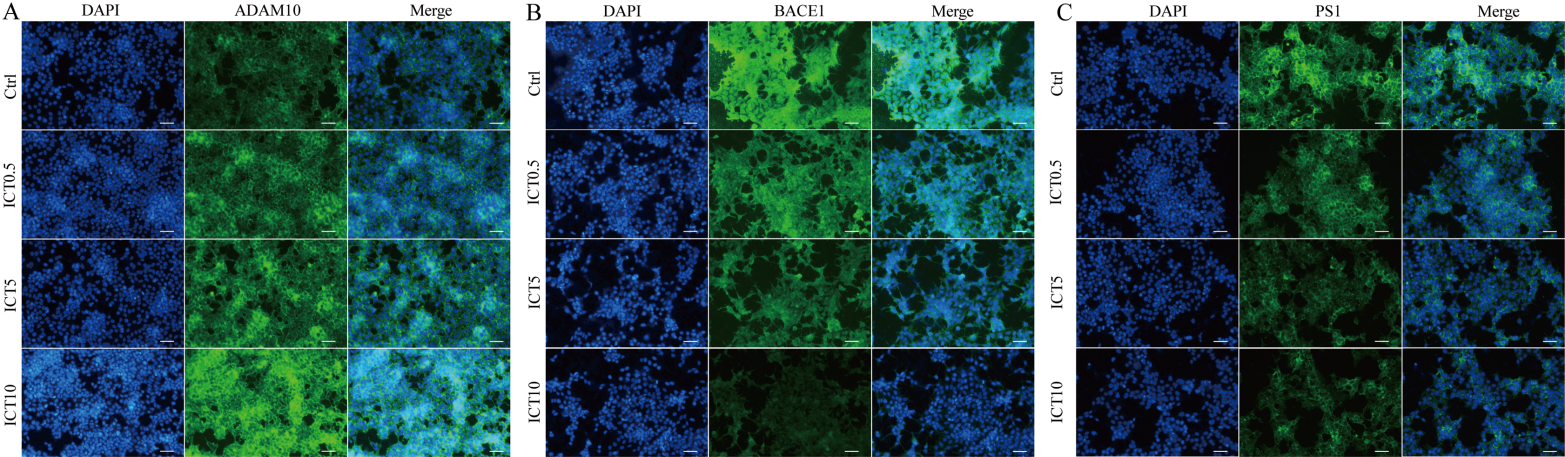
ICT reduced expression of BACE1 and PS1 and increased expression of ADAM10 in APP-PS1-HEK293 cells. Blue fluorescence represents the nuclei of APP-PS1-HEK293 cells, green fluorescence represents ADAM10, BACE1 and PS1, respectively. (A) ADAM10, (B) BACE1, (C) PS1. Scale bar is 20 μm, *n* = 3.

First, we focused on changes in expression of α-, β- and γ-secretase. We noted a significant increase in expression of ADAM10 in the ICT group compared with that in the control group (Fig. 4A; Fig. 5; Fig. 6A). We observed a significant decrease in BACE1 expression in the ICT group compared with that in the control group (Fig. 4B; Fig. 5; Fig. 6B). We documented a significant decrease in PS1 expression in the ICT group compared with that in the control group (Fig. 4C; Fig. 5; Fig. 6C). These results suggested that ICT could promote the non-amyloidogenic pathway, and inhibit the amyloidogenic pathway, of APP.

To verify our results, we detected some APP fragments. Compared with the control group, protein expression of sAPPβ and CTFβ was reduced significantly, whereas that of sAPPα increased. Finally, we found that protein expression of AβP_1–40_ and AβP_1–42_ in APP-PS1-HEK293 cells was lower in ICT-treated groups compared with that in the control group.

## Discussion

*Epimedium* is a genus of flowering plants in the family Berberidaceae. The main monomer component extracted from it, icariin (ICA), has been studied extensively. Zhang et al. (2014) used APPV717I transgenic mice to study the effect and mechanism of action of ICA on AβP production. They found that ICA (30 and 100 μmol/kg per day) could reduce AβP levels by decreasing expression of APP and BACE-1 (Zhang et al., 2014). Li et al. (2015) found similar regulatory mechanisms in a study of ICA on cognitive impairments induced by permanent occlusion of bilateral common carotid arteries (PO-BCCAs) in rats. ICA reduced the level of AβPs in the rat hippocampus subjected to PO-BCCAs by reducing expression of BACE1 and increasing expression of ADAM10 (Li et al., 2015). ICA also has a similar effect in triple-transgenic mice that are more able to mimic AD. Chen et al. used 3× transgenic-AD mice to investigate the neuroprotective properties of ICA. They found that ICA reduced deposition of AβP plaques in AD mice, and inhibited BACE1 expression.

The data stated above suggest that an extract of *Epimedium* had an inhibitory effect on BACE1 expression. Also, as a derivative of ICA, ICT, has a lower molecular weight and can penetrate the blood brain barrier readily (see Supplemental Material). Therefore, ICT has better drug-development capabilities than ICA. Previously, we demonstrated that ICT can improve memory impairment in a rat model of AD by reducing AβP accumulation (Feng et al., 2017).

In the present study, we used swAPP695 and PSEN1dE9 double gene-transfected HEK293 cells. APP-PS1-HEK293 cells show high expression of APP and PS1 in *swAPP695* and *PSEN1dE9*, and can increase expression of AβPs significantly. Hence, APP-PS1-HEK293 cells can be used as an AD model.

First, we used a BACE1 FRET kit to determine the IC_50_ of ICT for BACE1. ICT inhibited the activity of BACE1 and the IC_50_ of ICT was 5.70 ± 1.09 μM, similar to that of other flavonoids such as quercetin and apigenin (Sathya et al., 2012). Next, MTT and LDH assays were employed to detect the neuroprotective effects of ICT in APP-PS1-293 cells. We found that ICT had neuroprotective effects at 5 μM and 10 μM in APP-PS1-293 cells. Then, we detected the effect of ICT on the amyloidogenic pathway of APP in APP-PS1-HEK293 cells. APP has two pathways in transmembrane secretion: non-amyloidogenic and amyloidogenic. The non-amyloidogenic pathway mainly produces soluble sAPPα by α-secretase (ADAM10 and ADAM17) (Gupta & Goyal, 2016). Simultaneously, the CTFα of APP remains, and CTFα can be cleaved further by γ-secretase (PS1) to produce APP intracellular domain CTFγ (AICD) and p3 peptide (which does not aggregate). This metabolic pathway predominates in healthy status, and is required to maintain normal physiologic functions. Compared with the control group, the level of ADAM10 and sAPPα in the ICT group increased, suggesting that ICT promotes the non-amyloidogenic pathway. AβPs are produced via the amyloidogenic pathway. First, APP is secreted by BACE1 from the N-termini of AβPs to produce a soluble APP N-terminal fragment (sAPPβ) and a CTFβ. Then, cleavage is continued by γ-secretase, resulting in a series of AβPs of varying lengths (39–43 amino acids) and AICD. When the balance between AβP production (amyloidogenic pathway) and AβP clearance (non-amyloidogenic pathway) is broken off, it causes abnormal deposition of AβPs in the brain (Castro, Hadziselimovic & Sanders, 2019; Tiwari et al., 2019). In the present study, compared with the control group, expression of sAPPβ, BACE1, PS1, and CTFβ in the ICT group decreased, indicating that ICT inhibits the amyloidogenic pathway.

The major findings of our study were that ICT could reduce production of AβP_1–40_ and AβP_1–42_ through inhibition of expression of BACE1 and PS1 (which are the two most important enzymes in the amyloid metabolic pathway) and that expression of ADAM10 increased simultaneously (which is another cause of reduction of AβP levels). BACE1 and γ-secretase have been considered to be potential targets for new therapeutic agents that block AβP generation (Coimbra et al., 2018; Kumar et al., 2018), though several issues must be addressed urgently (Voytyuk, De Strooper & Chávez-Gutiérrez, 2018). Here, we showed that ICT can decrease AβP levels and could be neuroprotective by reducing AβP generation. Our previous in vivo study revealed that ICT can alleviate memory impairment in an AD model in rats (Feng et al., 2017). Taken together, ICT might be developed for AD therapy.

## Conclusions

Our results indicated that ICT, as a BACE1 inhibitor, inhibits APP secretion in APP-PS1-HEK293 cells by impeding the amyloidogenic pathway.

## Supplemental Information

10.7717/peerj.8219/supp-1 Supplemental Information 1 Full-length uncropped blots.

10.7717/peerj.8219/supp-2 Supplemental Information 2 Raw data.

10.7717/peerj.8219/supp-3 Supplemental Information 3 Icariin can pass the blood–brain barrier.

## Additional Information and Declarations

### Data Availability

The following information was supplied regarding data availability: The raw measurements are available in the Supplemental Files.
