# Supplementary material for: Icaritin, an inhibitor of beta-site amyloid cleaving enzyme-1, inhibits secretion of amyloid precursor protein in APP-PS1-HEK293 cells by impeding the amyloidogenic pathway
Source: PeerJ. 2019 Dec 10;7:e8219. doi: 10.7717/peerj.8219 (PMC6910110; doi:10.7717/peerj.8219)

**Full-length uncropped blots**

**Fig. 5. *Effects of ICT on protein expression of the APP metabolic pathway in APP-PS1-HEK293 cells.***

sAPPa

Ctrl ICT0.5 5 10


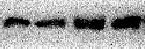


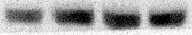


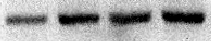


sAPPβ


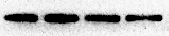


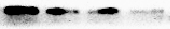


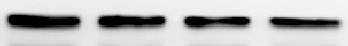


ADAM10


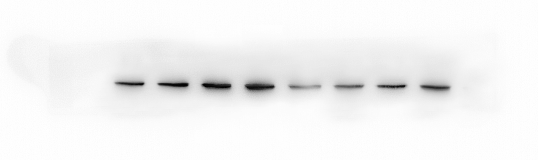


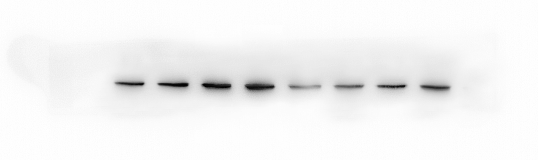

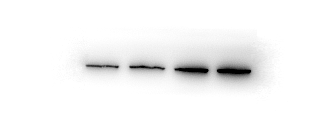


BACE1


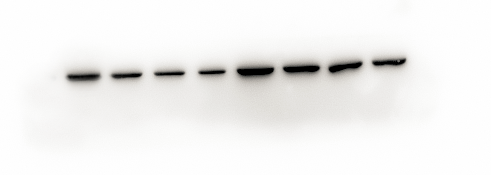


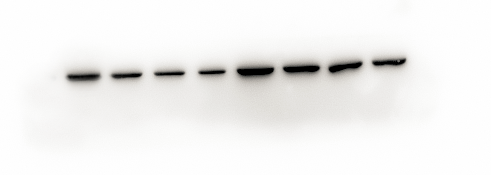


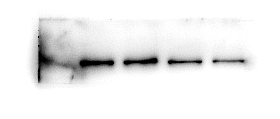


PS1


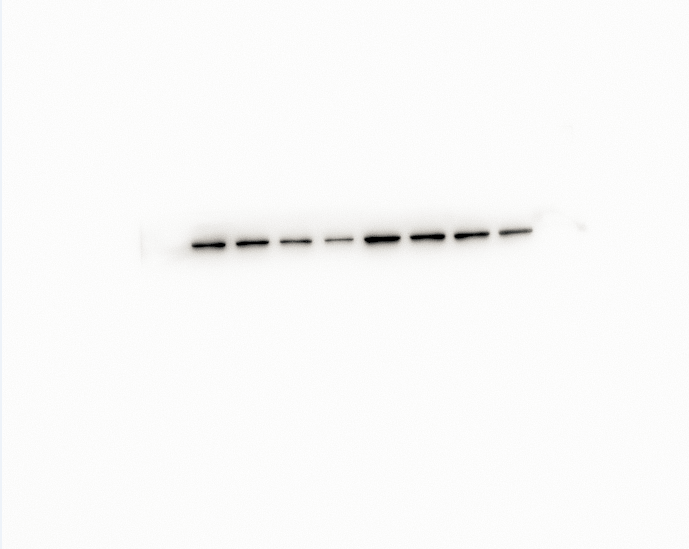


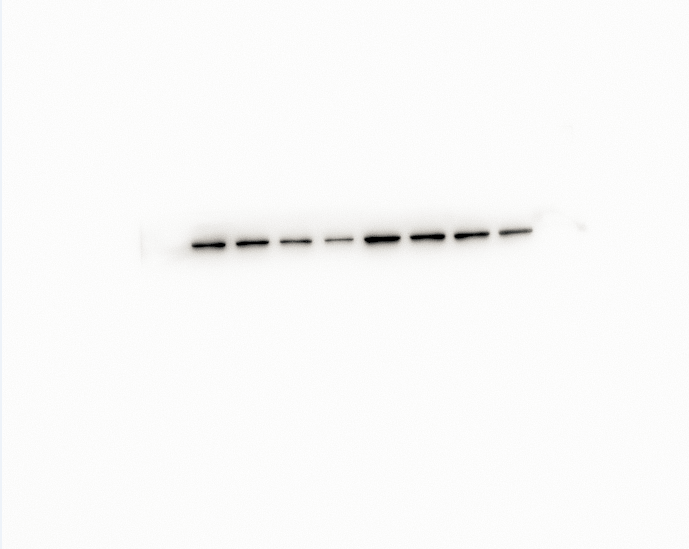


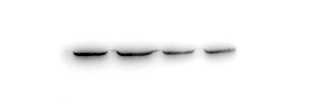


Aβ_1-42_


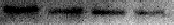


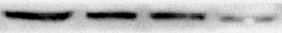


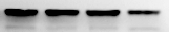


Aβ_1-40_


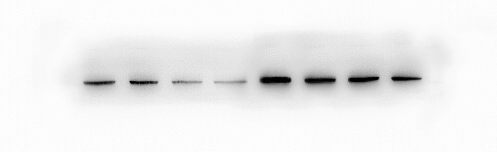


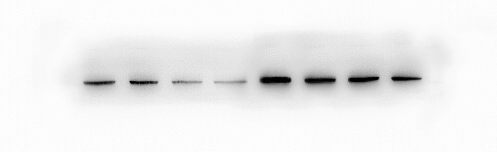


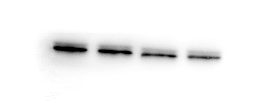


CTFβ


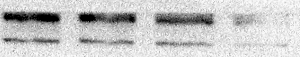

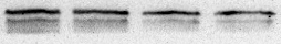


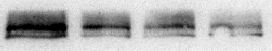


GAPDH


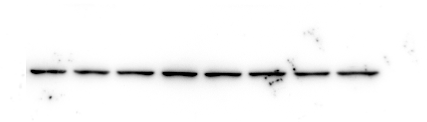


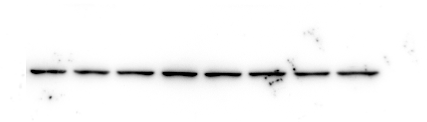


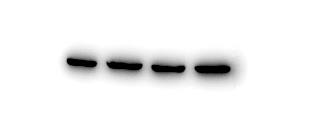

Supplement: Supplemental Information 1 [file peerj-07-8219-s001.docx]
