# Supplementary material for: Icaritin, an inhibitor of beta-site amyloid cleaving enzyme-1, inhibits secretion of amyloid precursor protein in APP-PS1-HEK293 cells by impeding the amyloidogenic pathway
Source: PeerJ. 2019 Dec 10;7:e8219. doi: 10.7717/peerj.8219 (PMC6910110; doi:10.7717/peerj.8219)
Supplement: Supplemental Information 3 [file peerj-07-8219-s003.docx]

**Icariin can pass the blood-brain barrier**

Icariin can pass through the blood-brain barrier and can be detected in rat brain tissue after intragastric administration. Below is the LC-MS result of Icariin (These results are provided by Key Laboratory of Basic Pharmacology and Joint International Research Laboratory of Ethnomedicine of Ministry of Education, Zunyi Medical University, thanks to Dr. Yanliu Lu for his work.)

A substance with a molecular weight of glucuronic acid added to the molecular weight of two icariin was estimated to be a metabolite of icariin in vivo (liver, brain) combined with glucuronic acid.

**Icaritin reference standard**


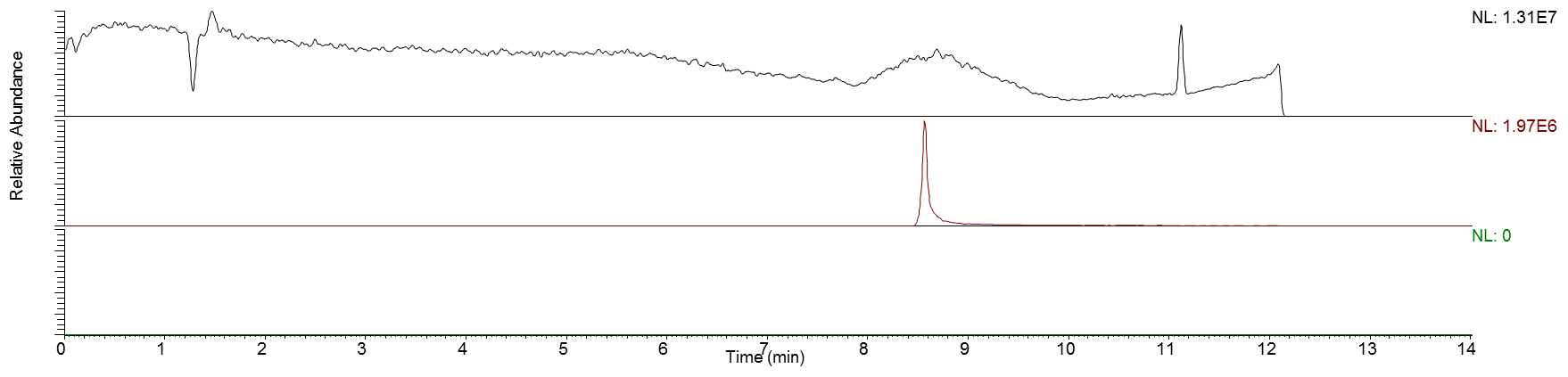


Icaritin

Icaritin + Glu acid

**Brain of Control group**
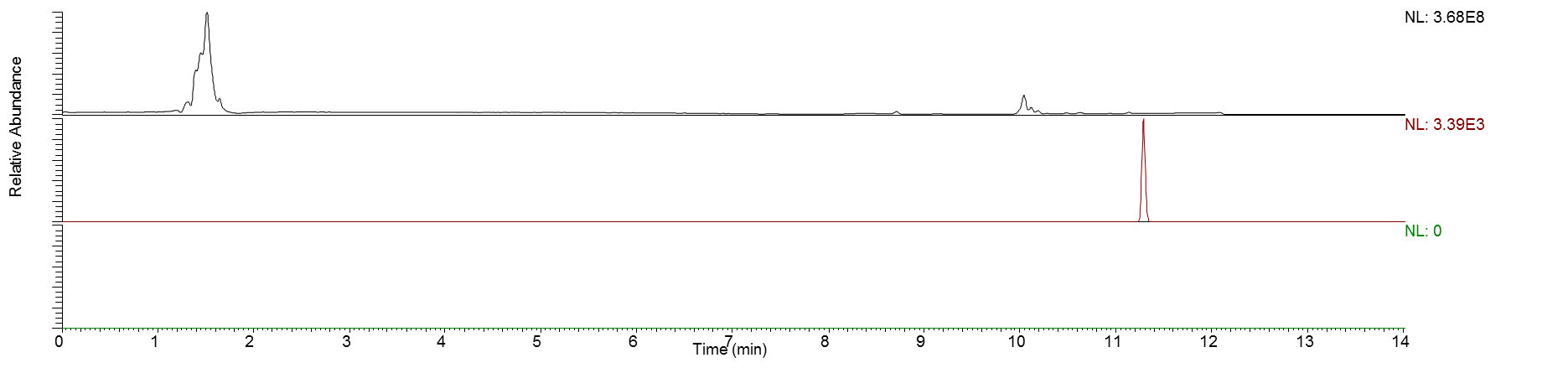


Icaritin + Glu acid

Icaritin

**Brain of Treatment group**


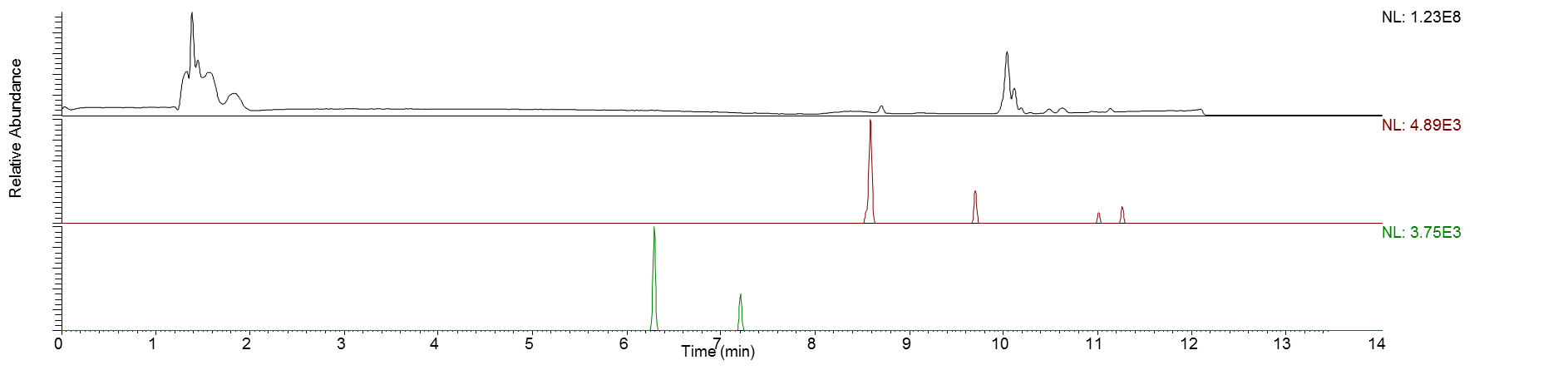


Icaritin

Icaritin + Glu acid
